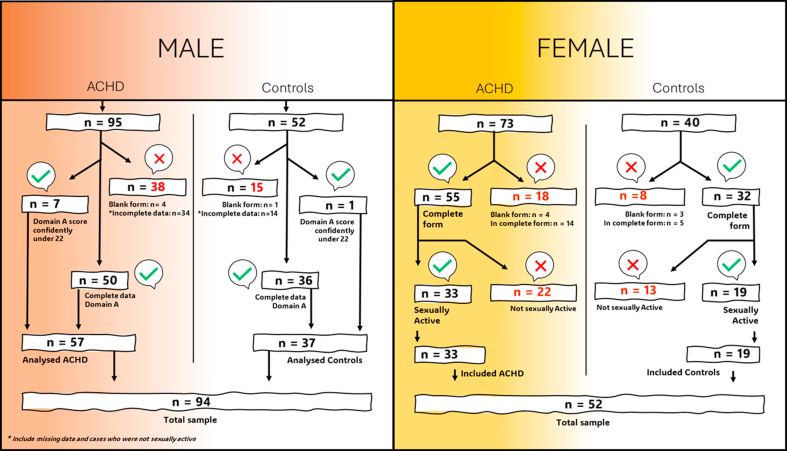# Sexual functioning after the age of 40 in adults with moderate or severe congenital heart disease^☆^

**DOI:** 10.1016/j.ijcchd.2026.100664

**Published:** 2026-02-27

**Authors:** Sandra Dalman-Skogby, Annika Bay, Christina Christersson, Joanna Hlebowicz, Zacharias Mandalenakis, Eva Goossens, Adrienne H. Kovacs, Liesbet Van Bulck, Koen Luyckx, Philip Moons, Camilla Sandberg, Bengt Johansson

**Affiliations:** a Department of Diagnostics and Intervention, Umeå University, Umeå, Sweden; b Region Västra Götaland, Sahlgrenska University Hospital, Children's Heart Center, Gothenburg, Sweden; c Department of Nursing, Umeå University, Umeå, Sweden; d Department of Medical Sciences, Cardiology, Uppsala University, Uppsala, Sweden; e Department of Cardiology, Skåne University Hospital, Clinical Sciences, Lund University, Lund, Sweden; f Department of Molecular and Clinical Medicine, Institute of Medicine, Sahlgrenska Academy, University of Gothenburg, Gothenburg, Sweden; g Centre for Research and Innovation in Care, Department of Nursing and Midwifery, Faculty of Medicine and Health Sciences, University of Antwerp, Antwerp, Belgium; h Department of Public Health and Primary Care, KU Leuven, Leuven, Belgium; i Department of Patient Care, Antwerp University Hospital, Antwerp, Belgium; j Equilibria Psychological Health, Toronto, Ontario, Canada; k Faculty of Psychology and Educational Sciences, KU Leuven, Belgium; l UNIBS, University of the Free State, Bloemfontein, South Africa; m Gothenburg Center for Person-Centered Care, University of Gothenburg, Sweden; n Department of Paediatrics and Child Health, University of Cape Town, South Africa; o Department of Community Health and Rehabilitation, Umeå University, Umeå, Sweden; p Department of Public Health and Clinical Medicine, Umeå University, Umeå, Sweden

**Keywords:** Heart defects, Congenital, Aging, Sexual health, Sexual dysfunction, Physiological, Dysfunction, Erectile

## Abstract

**Background:**

Sexual health and functioning are significant, yet often overlooked, components of psychosocial well-being of adults with congenital heart disease (ACHD). Prior research reports inconsistent findings, limited data on older adults with CHD, and few studies have compared patients with controls from the general population. This study investigated sexual functioning in middle-aged and elderly ACHD patients and compared it with controls.

**Methods:**

This was a Swedish sub-study of the international APPROACH-IS II project. Patients 40 years or older with moderate or severe CHD were recruited, along with controls from the general Swedish population. The design was case control. Erectile dysfunction (ED) in males was assessed using the International Index of Erectile Function. Sexual dysfunction (SD) in females was assessed using the Female Sexual Function Index.

**Results:**

Overall, 146 individuals were included in the study; 90 patients with CHD and 56 controls. Among males, the prevalence of ED was higher in patients (n = 57) than in controls (n = 37), (21% vs. 2.7%, *p* = 0.01). ED was associated with increasing age (OR = 1.11; 95% CI 1.03-1.20). Among females, no difference in the prevalence of SD was observed between patients (n = 33) and controls (n = 19) (27% *vs.* 30%, *p* = 0.76)

**Conclusion:**

Sexual dysfunction was more common in male patients than controls, whereas no differences were observed between female patients and controls. Given that approximately one in five males and one in four females with CHD reported sexual dysfunction, increased empirical and clinical attention is warranted.

## 1 Key message

### 1.1 What is already known on this topic?

Sexual dysfunction is more prevalent among ACHD patients. However, prior studies are limited by the lack of controls, and little is known about the experiences of women and patients of older ages.

### 1.2 What does this study add?

This study explored sexual dysfunction and erectile dysfunction in ACHD patients aged 40 years and older compared to controls. The study thereby contributed with an older patient cohort, comparison with controls, and the inclusion of female patients.

### 1.3 How may this study affect research, practice or policy?

The findings strengthen calls for increased empirical and clinical attention directed to the sexual health and function in the ACHD population. It may inspire further research into predictors and interventions for sexual dysfunction and prompt healthcare providers to identify referral pathways in clinical practice.

## 2 Introduction

Congenital heart disease (CHD) is the most common congenital malformation, with an incidence of approximately 1% of live births [1]. Heterogeneity in CHD is vast, encompassing a range of lesion complexity, interventional needs, and survival rates. Advances in medical care have led to increased survival, with certain CHD lesions now achieving a remarkable 98% survival rate into adulthood [2,3]. As a result, the adult congenital heart disease (ACHD) population is steadily growing [4], shifting the research focus from short-term survival to long-term concerns, such as heart failure, arrhythmias and need for reinterventions, but also less studied topics, such as quality of life, mental health and sexual health. Sexual health and functioning represent significant, yet often overlooked, factors in the psychosocial well-being of individuals with CHD. Although sexual health is a recognized aspect of quality of life, research on its implications for those with CHD remains insufficient [[5], [6], [7], [8]].

In males, research tends to focus on erectile dysfunction (ED), *i.e. the inability to attain or maintain an erection sufficient for satisfactory sexual performance,* that may have a profound impact on quality of life [9]. In the general population, the prevalence of ED increases with age, primarily affecting males older than 40 [10,11]. The causes of ED are multifactorial, ranging from psychological and neurogenic factors to endocrinological, vasculogenic, and drug-related causes [10]. A vascular aetiology is common, and ED has been shown associated with cardiovascular disease and all-cause mortality [11,12], potentially making the ACHD population vulnerable. Prior studies suggest that the prevalence of ED in males with CHD ranges from 10% to 43% [[7], [13], [14], [15], [17]]. One study reported that ED was more common in adults with CHD compared to normative data [7], whereas another study found no difference between those with CHD and historical controls [13]. To date, only one study compared adults with CHD to controls, in which sexual problems were less prevalent in the ACHD population; however, this study did not specifically investigate ED [6]. Given this limited knowledge foundation, additional studies are needed to compare the prevalence of ED between adults with CHD and controls. In addition, most previous research has mainly focused on individuals younger than 40 years [6,7,[13], [14], [15], [16], [17], [18]]. Since ED increases with age in the general population [10,11], there is a need for further research on older adults with CHD.

Sexual dysfunction refers to perceived problems in a wide spectrum of functions, with lack of desire being one of the most reported [19,20]. For females with CHD, sexual functioning may be compromised due to factors such as heart failure or medication side effects. Age-related changes, such as menopause and decreased lubrication, can also influence sexual health and functioning [21]. Previous research suggested that 15-25% of females with ACHD experience sexual dysfunction [7,16] and has also shown that females with ACHD are more likely than males to report not enjoying sex, feeling insecure about having sex, and not feeling aroused during sex [6]. Sexual difficulties being more prevalent among females has also been observed in the general population [22].

Given the still quite limited understanding of sexual functioning in adults with ACHD, this study investigated sexual functioning in older males and females with CHD, compared it with controls and investigated associated factors.

## 3 Methods

### 3.1 Patient sample

This is a Swedish sub-study of the international APPROACH-IS II project [23]. The sample used in the present study is drawn from the larger APPROACH-IS II project cohort, more specifically, the patients included from Sweden. Patients with ACHD were recruited from four Swedish university hospitals during 2021-2022. Inclusion criteria were ≥40 years of age, moderate or severe CHD diagnosis before age 10 years, literate and speaking Swedish. An exclusion criterion was the inability to make independent decisions.

Medical history was collected and was based on medical records and included age, CHD diagnosis, interventions, history of heart failure (yes/no as assessed by a cardiologist), ventricular dilation and function, New York Heart Association (NYHA) functional classification, pulmonary hypertension, history of arrhythmia, anti-arrhythmic medications, implantable cardioverter defibrillator, pacemaker, hypertension, diabetes mellitus and acquired cardiovascular disease. Data on height and weight, smoking status, marital status and educational level were extracted from surveys filled out by study participants. More details about the data collection in the larger study can be found in the study protocol [23].

In the present study, males and females were split into two different groups; here we refer to the sex assigned at birth.

### 3.2 Controls

The control subjects were recruited from the Swedish population registry based on age and sex, according to a prespecified algorithm to minimize selection bias. Recruitment of and data collection from control subjects occurred at Umeå University Hospital 2022. Eligible control subjects were asked to participate in the study by telephone. Medical history was based on surveys and included age, height, weight, smoking status, hypertension, diabetes mellitus, acquired heart disease, marital status and educational level.

### 3.3 Erectile function in males

The International Index of Erectile Function (IIEF) questionnaire was chosen to assess erectile function in male participants. It is a multidimensional instrument addressing erectile function as well as sexual functioning in males [24,25]. The IIEF is a self-administered questionnaire with 15 questions (items) regarding five different domains: Domain A, erectile function, Domain B, orgasmic function, Domian C, sexual desire, Domain D, intercourse satisfaction, and Domain E, overall sexual satisfaction. The questions are restricted to sexual activity during the four weeks before answering the questionnaire. Analysis of IIEF scores thus requires sexual activity during that period. We utilised domain A, erectile function (henceforth referred to as IIEF-EF) within the IIEF questionnaire to address erectile function. The IIEF-EF domain contains 6 questions addressing erectile function. Higher scores reflected better sexual functioning, and the highest possible IIEF-EF score is 30. A cut-off value below 22 is used to characterize individuals as having ED [24]. Scores are further subdivided as mild (score 17-21), moderate (score 11-16) or severe (score 6-10). Participants who indicated sexual inactivity during the last four weeks by reporting an item score of 0 were excluded from the analysis. Participants with incomplete IIEF-EF data were included in the analysis only if the available responses allowed for a confident classification under the established cut-off value, i.e. when a hypothetical maximal score on the unanswered questions still results in a total score below 22. Otherwise, participants with incomplete data were excluded from the analysis.

The IIEF questionnaire was completed by both male patients and controls.

### 3.4 Sexual functioning in females

To determine sexual function, we utilised the Female Sexual Function Index (FSFI), which is a multidimensional scale for assessing female sexual functioning during the last 4 weeks [26]. The scale includes six different domains: sexual desire, arousal, lubrication, orgasm, satisfaction, and pain, altogether 19 items. The total score range is 2-36 points, with a maximum score of 6.0 within each domain. The higher the score, the better the sexual functioning [27]. The total FSFI score can be used to distinguish individuals with sexual dysfunction. The different domains of the FSFI represent the primary components of sexual functioning in females, and individual domain scores can provide information on how the individual components contribute to sexual function.

We applied the suggested cut-off score of ≤26.55 to distinguish individuals with sexual dysfunction (SD) [26]. Participants who indicated sexual inactivity during the last four weeks by reporting an item score of 0 or participants with incomplete/blank FSFI data were not included in the analysis.

The FSFI questionnaire was complete by both female patients and controls.

### 3.5 Ethics

All study participants provided written informed consent for participation. The study was conducted in agreement with the Helsinki Declaration [28] and was approved by the Regional Ethics Review Board (Swedish registration number Dnr 2019-06247).

### 3.6 Statistics

Categorical variables were reported as numbers with percentages. Comparisons between groups were performed using the Chi-square test, Fisher's exact test, or Fisher-Freeman-Halton exact test. Continuous variables were reported as means with standard deviation. Continuous data were assessed for normality and compared using the Student's *t*-test or the Mann-Whitney *U* test.

The association between erectile dysfunction and independent variables in males was assessed using univariable and multivariable logistic regression. Regression analyses were reported with odds ratios (OR) and 95% confidence intervals (95% CI).

Associations with sexual functioning scores in females were assessed using the Chi-square test, univariate logistic regression, and univariate linear regression analysis. Logistic regression analyses were reported using odds ratio (OR) and 95% confidence intervals (95% CI).

Independent variables for association analysis included age, marital status, and CHD-specific characteristics, such as CHD complexity, NYHA class, arrhythmia diagnoses, and arrhythmia medication. Due to the lack of univariate associations, no multivariable models were applied.

The statistical analyses were performed using the IBM SPSS Statistics for Windows, version 28 (IBM Corp., Armonk, NY, USA). The null hypothesis was rejected on *p*-values <0.05.

## 4 Results

### 4.1 Overall inclusion

Overall, 146 individuals met full inclusion criteria, including sexual activity during the preceding four weeks and were thus included in the study, among which 90 were patients with CHD and 56 were control subjects (Supplementary Fig. 1).

### 4.2 Included males

A total of 57 males with ACHD and 37 male controls were included. A total of 38 male patients and 15 male controls were excluded (Supplementary Fig. 1).

The mean age of male ACHD patients was 56.5 ± 11.0 years (range, 40-78). Among male patients, coarctation of the aorta was the most common diagnosis (35%). The majority had moderately complex CHD (91%), and the majority were classified as New York Heart Association (NYHA) functional class I (88%). Heart failure was present in 19%, with ventricular dysfunction noted in 30%. Patients and controls did not differ in terms of sample demographics. (Table 2).

### 4.3 Included females

A total of 33 females with CHD and 19 female controls were included. A total of 40 female patients and 21 female controls were excluded (Supplementary Fig. 1).

The mean age of female ACHD patients was 51.9 ± 9.3 years (range, 40-74). Among female patients, coarctation of the aorta was the most frequent defect (24. %). Most had moderately complex lesions (82%), and most were classified as NYHA class I (76%). Patients and controls did not differ in terms of sample demographics. For further details, see Table 1, Table 2.

**Table 1:**
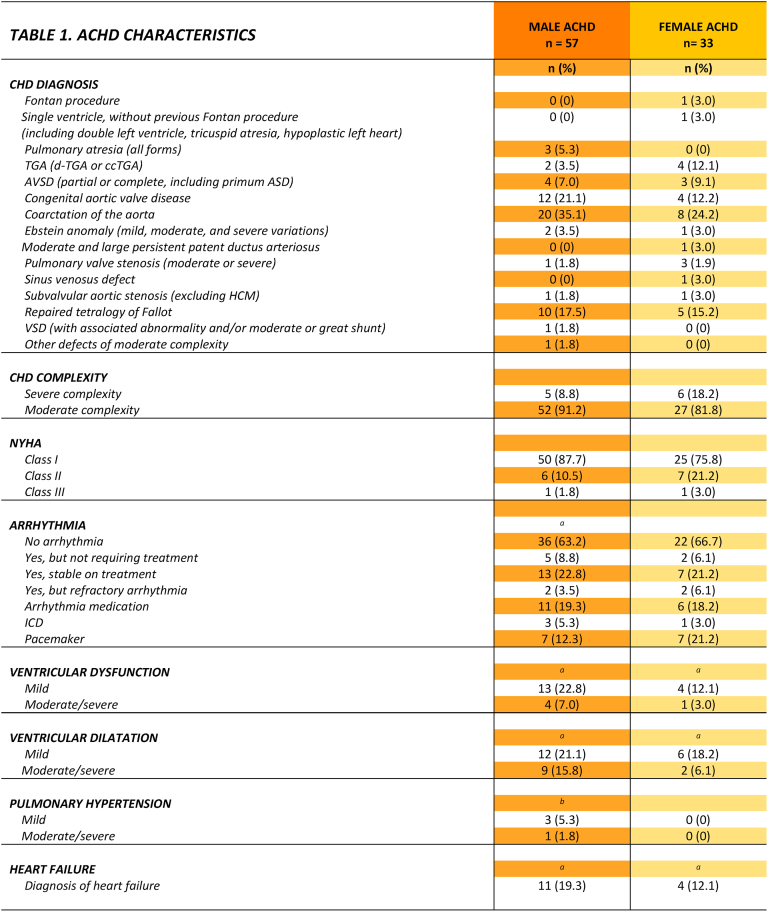
Achd characteristics.

NYHA =New York Heart Association Functional Class; TGA = Transposition of the great arteries; VSD = ventricular septal defect; AVSD = atrio ventricular septal defect; ICD=Implantable cardioverter defibrillator; ^a^ = Data missing for 1 participant^, b^ = Data missing for 2 participants.

**Table 2:**
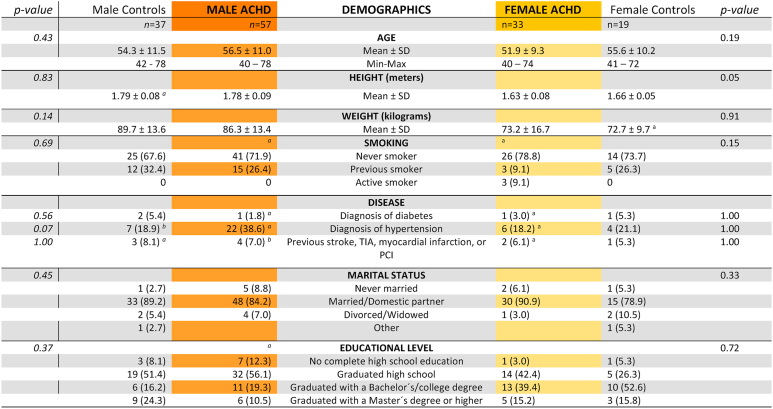
Sample demographics.

Continuous variables were non-normally distributed and analysed with the Mann-Whitney *U* test.

Categorical variables were analysed with the Chi-Square test, Fisher's exact test or Fischer-Freeman-Halton's exact test.

TIA = transient ischaemic attack; PCI = percutaneous coronary intervention. a = Data missing for 1 participant, b = Data missing for 2 participants.

### 4.4 Erectile dysfunction in males

The proportion of ED*,* defined as less than 22 points on the IIEF-EF (domain A), was higher among patients than controls (21% *vs*. 2.7%, *p* = 0.01). For individual domain scores, se Table 3, panel A.

**Table 3:**
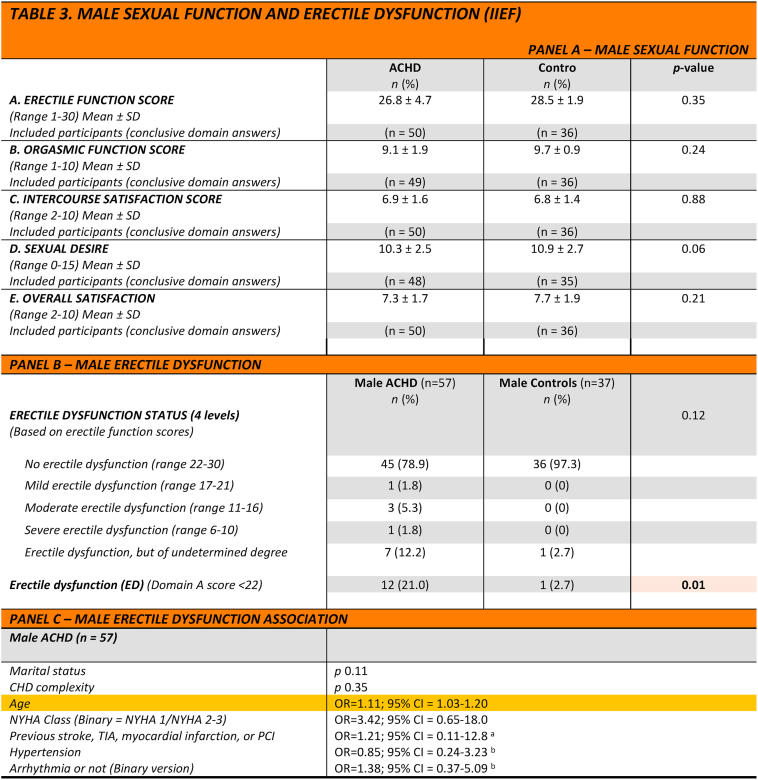
Male sexual function and erectile dysfunction (iief).

Analysis with Mann-Whitney *U* test, Chi-square test, Fisher's Exact test or Fischer-Freeman-Halton's exact test, and univariate/multivariate logistic regression analysis.

TIA = transient ischaemic attack; PCI = percutaneous coronary intervention, NYHA =New York Heart Association Functional Class Having a partner = married or domestic partner, b = Data missing for 1 participant, a = Data missing for 2 participants.

Eight participants (7 adults with CHD and one control) were included despite incomplete IIEF-EF data, since their score was determined to be below the cut-off value of 22 points despite incomplete data. However, the severity of their erectile dysfunction could not be reliably determined, and these participants were therefore reported as erectile dysfunction of undetermined degree. (Table 3, panel B).

ED was associated with increasing age for ACHD (OR = 1.11; 95% CI 1.03-1.20). No differences in ED status were found between patients with moderately *vs.* severely complex CHD, nor between NYHA classes or the presence/absence of hypertension, arrhythmia, marital status, or acquired heart disease (Table 3, panel C).

In a multivariable regression analysis, the complete sample, including both ACHD patients and controls, showed that ED was associated with age (OR = 1.13; 95% CI = 1.05-1.20) and congenital heart disease (OR = 12.2; 95% CI 1.3-113).

### 4.5 Sexual dysfunction in females

There were no differences in sexual dysfunction (≤26.55 points on the FSFI total score) between patients with CHD and controls (27% *vs.* 30% *p* = 0.76). Similarly, no differences were observed between patients and controls on any domain scores of the FSFI (Table 4). No associations were identified for sexual dysfunction or individual domain scores of the FSFI, including age, CHD complexity, NYHA class, arrhythmia and marital status (Supplementary Table 1).

**Table 4:**
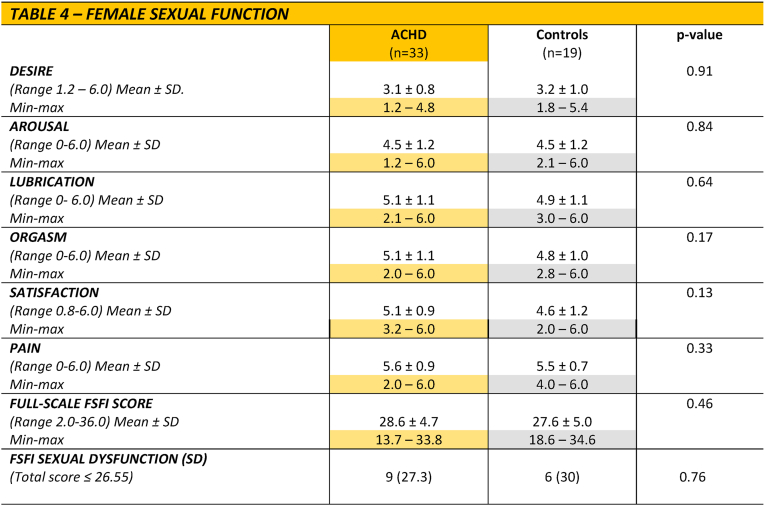
Female sexual function.

Analysis with the Man-Whitney *U* test for continuous variables, Fisher's Exact test, or Fischer-Freeman-Halton's exact test for categorical variables. ∗SD = Sexual dysfunction.

## 5 Discussion

Given the limited understanding of sexual functioning in adults with ACHD, this study seeks to examine sexual functioning in middle-aged and elderly males and females with CHD and compare the findings with a control group. We observed that ED was more prevalent in males with CHD than in controls. There was no obvious association with the complexity of the heart lesion. In females, sexual dysfunction was common but equally prevalent among patients and controls.

### 5.1 Erectile dysfunction in males

Prior studies suggest that the prevalence of ED in adults with CHD ranges from 10% to 43% [7,[13], [14], [15],^17^]. In studies that compared the prevalence of ED in adults with CHD to that in the general population, results have been mixed [7,13]. Our study identified 21% of males with ACHD experiencing ED, which is within the range of previous reports. However, compared to prior studies [7,[13], [14], [15],^17^], we investigated an older population, and since the prevalence of ED increases with age in both adults with CHD and in the general population [10,11,15,17,29,30], the reported 21 % ED was lower than expected. One limitation of these comparisons is the use of different definitions and cut-off values for ED. However, our reported prevalence of 21% in a cohort aged 40 and older is still low compared to, for example, 43% in the study by Fischer et al., which used the same ED cut-off as the present study but in a younger ACHD cohort, where only 27% were over 40 years of age [15].

Although the present study reports ED as more common among patients compared to controls, the reason behind this difference remains unclear. The causes of ED are multifactorial. A vascular aetiology is common, where endothelial dysfunction or reduced endothelial response is an important contributing factor, impairing vasodilatation [31]. Vascular disease or cardiovascular comorbidity, such as arterial hypertension or diabetes, might exacerbate endothelial dysfunction further and, thereby, also the occurrence of ED [31]. In the general population, studies highlight the link between cardiovascular disease and ED, as well as other conventional cardiovascular risk factors, such as smoking and obesity [10,[31], [32], [33], [34]]. However, this study detected no difference in the history of smoking or body weight between adults with CHD and controls.

### 5.2 Sexual dysfunction in females

Previous research on sexual health and functioning among females with CHD is limited, consistent with a 2013 survey completed by researchers and nurse specialists in which sexual functioning, pregnancy and gynaecological issues received the lowest possible priority scoring [35]. This priority has been reflected in published work over the past decade, with publishing, to our knowledge, of only two studies on sexual health and functioning in females with CHD [7,16].

This is one of the first studies to compare sexual health and functioning in females with CHD to controls. We utilised the FSFI to assess sexual health and functioning among females with CHD. In the present study, just above one fourth of patients had SD, which aligns with previous reports with proportions ranging from 15 to 25% [7,16]. The present study also aligns with results from the general population, where sexual problems in females are estimated to range between 25 and 63 % [22].

Looking at individual domain scores among ACHD patients, sexual desire had the lowest scores. This is in line with previous literature on the general population, where lack of desire is one of the most frequently reported sexual problems in females [19,20] but also in previous studies in females with ACHD [7].

The present study showed no significant differences in the prevalence of SD or individual domain scores of the FSFI between ACHD patients and controls. In the only prior case/controls study performed by Moons and colleagues [6], sexual problems were reported as less prevalent in the ACHD population compared to controls [6]; however, that study did not utilise the FSFI, but the CHD-TAAQOL. Comparing our results to previous studies utilising the FSFI, one study reported worse FSFI scores among females with CHD compared to normative data [7].

### 5.3 Methodological considerations

This is one of the first studies to compare sexual health and functioning in females with CHD to controls and is a valuable addition to the literature on this topic.

The main strengths of the present study are the CHD study population, which is ≥ 40 years old and recruited in multicentre mode, and the control subjects, which allow for comparisons with the general population. It is also a strength that the study applies validated instruments to assess sexual health and function. However, a few limitations ought to be considered when interpreting the results.

First, the sample size is small. Reasons for the small sample size include, per protocol, the exclusion of participants not being sexually active during the four weeks before completing the form. This exclusion is standard practice; however, it is unfortunate and affects the sample size and statistical robustness, potentially risking underestimating the issue of SD in this population, since we are only assessing SD in sexually active individuals. Second, 8 male participants (7 patients and 1 control) were included for analysis despite incomplete answers within domain A of the IIEF form. Participants with incomplete IIEF-EF data were included only when the available responses allowed a confident classification below the established cut-off value, even under the most conservative assumptions. This approach represents a deliberate trade-off between including incomplete data/accepting uncertainty in terms of severity and avoiding underestimation of the prevalence of erectile dysfunction. ED severity was not assessed and reported as erectile dysfunction of undetermined degree. Third, since such data was unavailable, the present study did not investigate the association between ED/SD and medication. Fourth, survivorship bias should be considered as a significant proportion of patients may have died before the age of 40 years. It is also important to consider that a significant group of patients with severe CHD have not yet reached high age, since they did not generally survive until the mid-nineties. Fifth, the present study and its cohorts are based on the sex provided at birth and does not consider gender identity or sexual orientation.

## 6 Conclusion

ED in males with ACHD ≥40 years of age was 21%, more than seven times higher compared to controls. Erectile dysfunction was associated with older age and having a CHD lesion. On the other hand, comorbidity, functional class, or other factors related to CHD did not affect the risk for ED.

The present study showed no difference in sexual health and functioning between females with CHD and controls, but more than one out of four female ACHD patients and controls had SD. No associations for SD could be identified.

Given the high proportion of sexual difficulties reported by patients in this study, increased empirical and clinical attention is needed.

## CRediT authorship contribution statement

**Sandra Dalman-Skogby:** Writing – review & editing, Writing – original draft, Visualization, Resources, Methodology, Investigation, Formal analysis, Data curation, Conceptualization. **Annika Bay:** Writing – review & editing, Resources, Investigation. **Christina Christersson:** Writing – review & editing, Resources, Investigation, Funding acquisition, Data curation. **Joanna Hlebowicz:** Writing – review & editing, Resources, Investigation, Funding acquisition, Data curation. **Zacharias Mandalenakis:** Writing – review & editing, Resources, Investigation, Data curation. **Eva Goossens:** Writing – review & editing, Resources, Project administration, Investigation, Data curation, Conceptualization. **Adrienne H. Kovacs:** Writing – review & editing, Resources, Investigation, Data curation, Conceptualization. **Liesbet Van Bulck:** Writing – review & editing, Resources, Investigation, Data curation, Conceptualization. **Koen Luyckx:** Writing – review & editing, Resources, Investigation, Data curation, Conceptualization. **Philip Moons:** Writing – review & editing, Supervision, Resources, Project administration, Methodology, Investigation, Funding acquisition, Data curation, Conceptualization. **Camilla Sandberg:** Writing – review & editing, Resources, Project administration, Investigation, Conceptualization. **Bengt Johansson:** Writing – review & editing, Writing – original draft, Supervision, Resources, Project administration, Methodology, Investigation, Funding acquisition, Formal analysis, Data curation, Conceptualization.

## Financial support

This study was funded by Regional Research Support in South Swedish Health Care Region, Skåne University Hospital funds (JH), the Swedish Heart and Lung Association, grant IDs 20190525 and 20240188 (JH, BJ), Greta and Johan Kock Foundations (JH), Anna-Lisa and Sven Eric Lundgren Foundation for Medical Research (JH), Visare Norr (BJ), Umeå University (BJ) and Region Västerbotten (BJ). Funders had not active part in carrying out the research or in the writing of the manuscript.

## Declaration of competing interest

The authors declare the following financial interests/personal relationships which may be considered as potential competing interests: Bengt Johansson reports financial support was provided by Swedish Heart and Lung Association. If there are other authors, they declare that they have no known competing financial interests or personal relationships that could have appeared to influence the work reported in this paper.

## Figures and Tables

**figs1:**