# Supplementary material for: Sexual functioning after the age of 40 in adults with moderate or severe congenital heart disease
Source: Int J Cardiol Congenit Heart Dis. 2026 Feb 27;24:100664. doi: 10.1016/j.ijcchd.2026.100664 (PMC13000524; doi:10.1016/j.ijcchd.2026.100664)
Supplement: Multimedia component 1 [file mmc1.docx]

| **SUPPLEMENTARY TABLE 1 – FEMALE SEXUAL FUNCTION ASSOCIATIONS** | | | | | | | | |
| --- | --- | --- | --- | --- | --- | --- | --- | --- |
|  | Linear and logistic regression analysis  Chi-square and Fishers exact test | | | | | | |  |
|  | **Dependent variables** | | | | | | | |
|  | DESIRE  SCORE | AROUSAL SCORE | LUBRICATION SCORE | ORGASM SCORE | SATISFACTION SCORE | PAIN  SCORE | FULL-SCALE SCORE | SEXUAL DYSFUNCTION |
| **Independent variables** | *Unstandardised coefficient*  *(Standard error)* | | | | | | | *OR = odds ratio*  *CI = confidence interval*  *p-value* |
| Age | - 0.01 (0.02) | - 0.01 (0.02) | - 0.03 (0.02) | - 0.02 (0.02) | 0.01 (0.02) | OR: 0.97 95% CI = 0.89 – 1.07 * | 0.05 (0.09) | OR: 1.00 95% CI = 0.92 – 1.09 |
| CHD complexity | 0.21 (0.39) | - 0.39 (0.57) | - 0.05 (0.49) | - 0.25 (0.51) | - 0.16 (0.42) | - 0.11 (0.41) | - 0.14 (2.15) | OR: 3.50 95% CI = 0.56-22.03 |
| NYHA Class | - 0.56 (0.34) | - 0.62 (0.50) | - 0.36 (0.44) | - 0.76 (0.44) | - 0.37 (0.38) | - 0.43 (0.36) | - 3.10 (1.86) | OR: 1.90 95% CI = 0.35-10.40 |
| Divorced/widow ** | 1.12 (0.88) | 1.16 (1.27) | 0.89 (1.11) | 0.88 (1.16) | 0.49 (0.97) | 0.41 (0.94) | 4.96 (4.84) | p=1.0 *Chi-square test including Marital status and sexual dysfunction.* |
| Never married ** | - 0.22 (0.63) | - 0.94 (0.91) | - 0.46 (0.80) | - 0.32 (0.83) | - 0.09 (0.69) | 0.41 (0.67) | - 0.99 (3.48) |  |
| Arrhythmia or not | - 0.19 (0.32) | - 0.84 (0.44) | - 0.18 (0.40) | - 0.44 (0.41) | - 0.44 (0.34) | - 0.20 (0.34) | - 2.29 (1.71) | OR: 3.75 95% CI = 0.75 – 18.70 |
| Arrhythmia medication or not | - 0.16 (0.39) | - 0.82 (0.55) | - 0.56 (0.45) | - 0.73 (0.50) | - 0.56 (0.41) | - 0.52 (0.40) | - 3.34 (2.07) | OR: 3.50 95% CI = 0.56 – 22.03 |
| Continuous dependent variables are analysed with linear regression. Categorical dependent variables are analysed with logistic regression or Chi-Square test /Fisher's exact test.  * Dependent variable categorised due to non-normal distribution  ** Compared to married/living with a partner | | | | | | | | |
